# Effect of Total Flavonoids of *Oxytropis falcata* Bunge on the Expression of p-JAK1-and p-STAT1-Related Proteins in Idiopathic Pulmonary Fibrosis

**DOI:** 10.1155/2020/2407239

**Published:** 2020-08-28

**Authors:** Yan-jun Wang, Yang Li, Xue-lin Wang, Xin-ze Li, Yan-wen Chen, Ling-ling Yang, Hai-xia Ming

**Affiliations:** ^1^Gansu University of Chinese Medicine, Lanzhou 730000, China; ^2^Provincial-Level Key Laboratory for Molecular Medicine of Major Diseases and the Prevention and Treatment with Traditional Chinese Medicine Research in Gansu Colleges and Universities, Lanzhou 730000, China; ^3^Laboratory of Chinese Medicine Pharmacology and Toxicology, Lanzhou 730000, China; ^4^Gansu University of Traditional Chinese Medicine, Lanzhou 730000, China

## Abstract

**Objective:**

The study aimed to explore the effect of total flavonoids of *Oxytropis falcata* Bunge (FOFB) on the expression of p-JAK1/p-STAT1 and SOCS3 proteins in idiopathic pulmonary fibrosis (IPF).

**Methods:**

Rats model with IPF was established by one-off intratracheal injection of bleomycin (BLM, 5 mg/kg). After 14 days, the same volume of low dose (100 mg/kg), medium dose (200 mg/kg), and high dose (400 mg/kg) of FOFB and prednisolone acetate (20 mg/kg) as positive control drugs, as well as normal saline, were orally administered to rats once a day for 28 consecutive days. Subsequently, the degree of fibrosis and alveolitis in rat lung tissue was observed, respectively, by HE and Masson staining. Further more, observing the ultrastructure of lung tissue by transmission electron microscopy (TEM), the detection of JAK/STAT pathway related indicators including p-JAK1, p-STAT1, and SOCS3 with immunohistochemistry and SOCS3 with real-time PCR (RT-PCR) was performed.

**Results:**

Compared with the BLM group, the degree of alveolitis and fibrosis improved significantly, and the expression of p-JAK1 and p-STAT1 decreased; conversely, the expression of SOCS3 increased in the treatment group.

**Conclusion:**

IPF causes high expression of p-JAK1 and p-STAT1 and low expression of SOCS3. FOFB can play a role in the treatment of IPF via upregulating SOCS3 and downregulating p-JAK1 and p-STAT1.

## 1. Introduction

Idiopathic pulmonary fibrosis (IPF), characterized by progressive dyspnea, is a chronic progressive interstitial lung disease [1]. After diagnosis, without a lung transplant, the median survival rate is 3–5 years, and the patient eventually dies from respiratory failure [2]. Nowadays, the specific etiology and pathogen are still unknown [3]. Most studies suggest that the occurrence of IPF is characterized by repeated subclinical damage to the alveolar epithelium [4–6]. After the lung tissue is damaged, the alveolar epithelium cell is activated. On the one hand, chemokines are released to recruit fibroblasts (FB) and a large number of FB subsequently can produce extracellular matrix (ECM). Besides, FB can also be transformed into myofibroblasts (MFB) under the induction of fibrogenic factors (such as transforming growth factor-*β*) generating ECM [7]. The excessive deposition of ECM accelerates the occurrence of pulmonary fibrosis. Several key signaling pathways play important role in the regulation process, including JAK/STAT, NF/*κ*B, and MAPK signaling pathways [8]. Among them, the JAK/STAT signal pathway is most closely related to the occurrence of IPF [9].

JAK protein kinas has a molecular weight of 120–130 kDa. So far, four members, JAK1, JAK2, JAK3, and TYK2, have been identified, which play important and diverse functions in hematopoietic, inflammatory, and immune responses. Among them, JAK1 is the most expressed JAK kinase, which is related to the signal transduction process of many cytokines and involved in inflammation and immune deficiency. STATs have a molecular weight between 79 and 113 kDa, and members of seven STAT protein families including STAT1, STAT2, STAT3, STAT4, STAT5A, STAT5B, and STAT6 have been found. STAT1 is the earliest discovered and cloned member of the STAT family [10]. Animal experiments have shown that STAT1 is abnormally expressed in bleomycin- (BLM-) induced IPF mice [11]. JAK1 is inactive before being exposed to cytokines, but the binding of cytokines to their receptors induces their own activation through transphosphorylation (i.e., formation of p-JAK1). Activated p-JAK1 phosphorylates its downstream signaling protein STAT1 (i.e., forming p-STAT1), causing them to dissociate from the receptor and transport to the nucleus. Then, p-STAT1 acts as a transcription factor in the nucleus to induce transcription of the target gene [12]. The SOCS protein family (especially SOCS3) is the most widely known negative regulator of the JAK/STAT pathway [13]. In addition, SOCS3 also has the ability to directly inhibit JAK kinase activity [14].

Total flavonoids of *Oxytropis falcata* Bunge (FOFB), extracts of *Oxytropis falcata* Bunge, are the major active components. Studies have confirmed that it has multiple pharmacologic actions, including being antitumor, being antioxidant, improvement of insulin resistance, protection against UV damage, enhancement of immune cofunction, being anti-inflammatory, and analgesia [15, 16]. Among them, the anti-inflammatory effect is the most significant. Other studies have shown that FOFB can reduce the expression of profibrosis factors such as typeIand type III collagen, inhibit TGF-*β*1-induced human renal tubular epithelial cell proliferation, and slow down the progress of fibrosis [17, 18]. Inflammation plays an irreplaceable role in the process of IPF, and FOFB has a significant anti-inflammatory effect. However, whether FOFB can exert IPF-preventing effects by affecting the expression of inflammatory factors, such as p-JAK1/p-STAT1 and SOCS3, is still unclear.

In the current study, to clarify the specific effects of FOFB on the Inflammation related protein including p-JAK1, p-STAT1, and SOCS3, rats models with IPF were established with BLM. This study aims to find specific targets that block the JAK-STAT signaling pathway in inflammation and provide an experimental basis for the treatment of IPF.

## 2. Material and Methods

### 2.1. Animals

Sixty adult specific pathogen-free animal (SPF) Sprague Dawley (SD) rats in half genders (200 ± 20 g) were provided by the Experimental Animal Center of Gansu University of Chinese Medicine. The environmental temperature was controlled at 25 ± 2°C, humidity was maintained at 45%–55%, and circadian rhythm was 12:12 h dark/light. All rats were fed with sterilized diet and water. Animal welfare was in compliance with the principles and procedures of Health Guide for the Care and Use of Laboratory Animals. The study protocols were approved by the Animal Care and Use Committee of Gansu University of Chinese Medicine.

### 2.2. Chemicals and Reagents

*Oxytropis falcata* Bunge was purchased from Tianzhu County Tibetan Medicine Development Institute. Total flavonoids of *Oxytropis falcata* Bunge (FOFB) were extracted and identified by Lanzhou University. The extraction method was ethanol immersion. The specific process included putting 5000 grams of medicinal materials in a 5000 mL round-bottom flask, immersing in 10 times the amount of absolute ethanol, soaking for 3 days, repeating 3 times, taking the filtrate, and spin-drying to obtain the product. Bleomycin was obtained from Nippon Kayaku (Tokyo, Japan). Prednisone was purchased from Zhejiang Xianju Pharmaceutical Co., Ltd., Zhejiang, China. Rabbit antibodies of p-JAK1/p-STAT1 and SOCS3 were purchased from Abcam plc, Cambridge, UK.

### 2.3. Grouping and Medications

Rats were randomly divided into six groups including blank (control) group, model (BLM) group, low-dose FOFB (100 mg/kg) group, middle-dose FOFB (200 mg/kg) group, high-dose FOFB (400 mg/kg) group, and prednisone acetate group (20 mg/kg) with 10 rats in each group. After intraperitoneal anesthesia with 3% pentobarbital sodium (30 mg/kg), the rats were fixed in supine position. With the help of laryngoscope, BLM (5 mg/kg) was injected into the trachea. The control group was given the same volume of physiological saline. After the injection, the animal was straightened and immediately rotated left and right to distribute the lung fluid evenly. After 14 days of modeling, rats in each group were given 2 mL of corresponding dose of drugs by gavage once daily. All rats were sacrificed on day 28.

### 2.4. Histopathology

After sacrifice, part of the left lung tissue was fixed in 4% paraformaldehyde, subsequently embedded in paraffin, serially sectioned, and stained with hematoxylin and eosin (H&E) or Masson's trichrome staining kit (Sigma-Aldrich) following manufacture's protocol. Histopathologic score was graded by specialized histopathologists blindly referring to Szapiel's semiquantitative grading system to divide into 4 grades and described the fibrosis score. The slices stained by Masson's trichrome were picked-up pictures by camera of microscope system and processed by Image-Pro plus version 6.0 for Windows.

### 2.5. Transmission Electron Microscope

Parts of lung tissue specimen was fixed by 2.5% glutaraldehyde, followed by 1% osmic acid postfixation, routinely dehydrated, and embedded in epoxy resin. It was then sliced into ultrathin sections and observed by a transmission electron microscope and images by AMT camera system were saved.

### 2.6. Immunohistochemistry

Lung tissue was sliced and dewaxed to water (xylene I 15 min, xylene II 15 min, xylene III 15 min, absolute ethanol I 5 min, absolute ethanol II 5 min, 85% alcohol 5 min, 75% alcohol 5 min, and distilled water). After repairing with citric acid antigen repair buffer, add 3% hydrogen peroxide to incubate. After blocking at room temperature for 30 minutes and adding rabbit polyclonal anti-p-JAK1 (1 : 200 vol/vol, Abcam), anti-p-STAT1 (1 : 100 vol/vol, Abcam), and anti-SOCS3 (1 : 100 vol/vol, Abcam), put it in a wet box at 4°C refrigerator overnight. After washing with PBS, adding secondary antibody (goat anti-rabbit) (1 : 200), DAB color development, and observing under the microscope, the reaction was terminated when it showed brown. Then, hematoxylin counterstained and returned to blue, and the neutral resin sealed.

### 2.7. Reverse Transcription Polymerase Chain Reaction (RT-PCR)

Total RNA extraction and cDNA synthesis were performed according to kit instructions. Semiquantitative PCR primers were designed according to SOCS3 cDNA sequence in NCBI. The SOCS3 primer was 5′-TCTTTACCACCgACggAACC-3′ and 5′-gTACCAgCgggATCTTCTCg-3'. The internal control GAPDH primer was 5′-AgTgCCAgCCTCgTCTCATA-3′ and 5′-TTgTCACAAgAgAAggCAgC-3'. For the PCR reaction, 2 *μ*l of cDNA, 2 *μ*l of primer, and 10 *μ*l of 2 × PCR Master Mix were added to 20 *μ*l of dd H_2_O. The reaction conditions were as follows: 95°C denaturation for 5 min, 95°C denaturation for 10 s, 60°C annealing for 20 s, 72°C extension for 20 s, and 42 cycles.

### 2.8. Statistical Analysis

All data conforming to normal distribution were presented as mean ± standard deviation (SD), and those not conforming to normal distribution were represented by median and interquartile spacing. Data were analyzed by one-way analysis of variance (ANOVA) of the SPSS 24.0 software. Statistical difference was considered significant when *p* value is less than 0.05.

## 3. Results

### 3.1. FOFB Ameliorated the Progress of Inflammation and Fibrosis in BLM-Induced IPF in Rats

**Figure 1:**
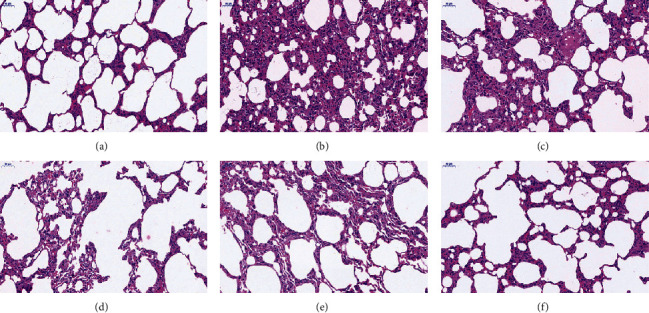
H&E staining of lung tissues (200x). (a) Blank (control) group. (b) BLM group. (c) Low-dose FOFB group. (d) Middle-dose FOFB group. (e) High-dose FOFB group. (f) Prednisone group.

We assessed the extent of inflammation in the lung tissue with HE staining. As shown in Figure 1. The picture of control group showed the bronchial and alveolar structures were intact, the alveolar septum was not thickened, and occasionally inflammatory cell infiltration was observed under the microscope. Sample of BLM group shows most of the alveolar structures collapsed and fused, and the interstitial cells increased, showing obvious fibrosis; the alveolar septa were significantly widened and thickened, and the infiltration of inflammatory cells increased significantly. Compared with the BLM group, low-dose FOFB group showed no significant difference; middle-/high-dose FOFB group and prednisone group could improve the destruction of alveolar structure and reduce the area of alveolar space and inflammatory cell infiltration, especially in the FOFB high-dose group and prednisone acetate group.

Szapiel score is shown in Table 1. The treatment group with middle-/high-dose FOFB and prednisone groups were lower than BLM group (*p* < 0.05).

**Figure 2:**
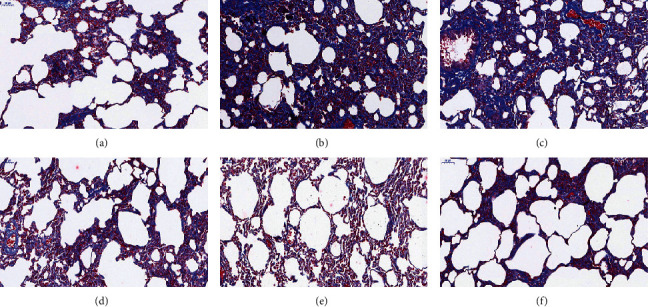
Masson's trichrome staining of lung tissues (200x). (a) Blank (control) group. (b) BLM group. (c) Low-dose FOFB group. (d) Middle-dose FOFB group. (e) High-dose FOFB group. (f) Prednisone group.

We evaluate the degree of fibrosis of the lung tissue by Masson's trichrome, as shown in Figure 2. In the blank group, there was no obvious change in the lung tissue of the rats. The collagen layer of the bronchial wall is thin, and a small number of light blue collagen fibers were distributed in the alveolar wall. In the BLM group, a large number of blue, cord-like, and lamellar collagen fibers stained around the bronchus and small blood vessels and in the lung interstitium were diffusely distributed. And there were a large number of alveolar fusions. The area of collagen deposition was decreased in the middle-/high-dose of FOFB and prednisone acetate groups, especially in the latter two groups.

According to the scoring standard of Masson staining, the results are shown in Table 2. There was a significant difference between the highest score of the BLM group and the lowest score of high-dose FOFB and prednisone acetate group (*p* < 0.01).

### 3.2. FOFB Alleviated the Abnormalities of Lamellar Number and Structure in IPF Rats Induced by BLM

**Figure 3:**
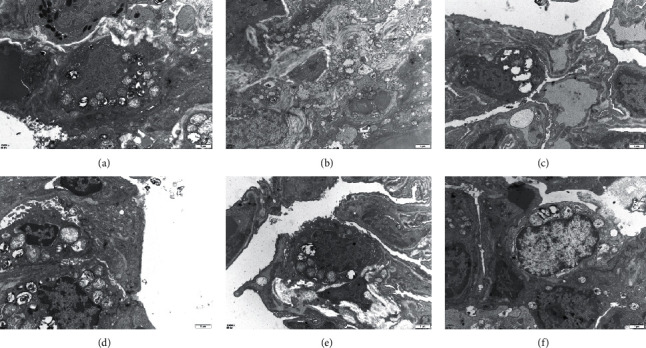
Observation of the structure, quantity, and arrangement of lamellar bodies with ultrastructure of lung tissue (12000x). (a) Blank (control) group. (b) BLM group. (c) Low-dose FOFB group. (d) Middle-dose FOFB group. (e) High-dose FOFB group. (f) Prednisone group.

Lamellar bodies (LBs) are organelles present in type II alveolar epithelial cells (AECII) and are closely related to the secretion of alveolar surfactant. Specific lamellar bodies are the most direct method to identify whether the secretory function of type AECII is normal. In our experiments, as shown in Figure 3, it was observed that the LBs of the blank group had a normal structure, with a large number, and neatly arranged; while the number of model groups was significantly reduced, the structure was incomplete and the arrangement was disordered. Compared with the BLM group, the number of LBs in the treatment with FOFB and prednisone acetate groups increased, the structure tended to be normal, and the arrangement tended to be neat.

### 3.3. Effects of FOFB on the Expression of p-JAK1, p-STAT1, and SOCS3 with IPF in Rats

**Figure 4:**
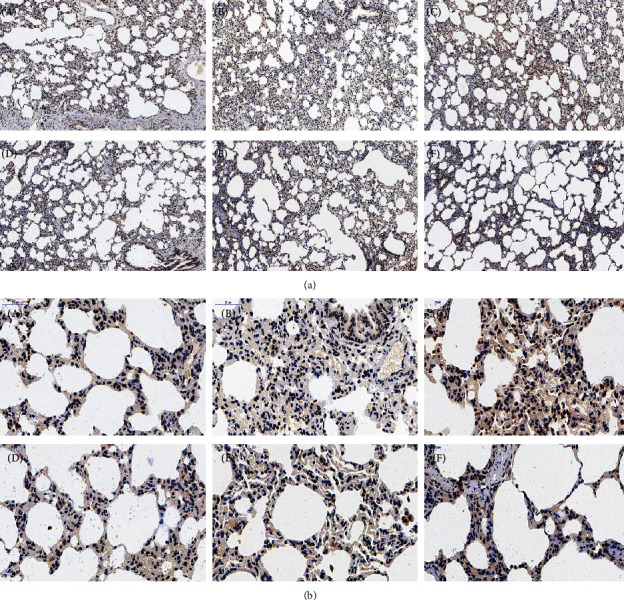
Observation of p-JAK1 by immunohistochemical staining ((a):100x; (b):400x). A: blank (control) group; B: BLM group; C: low-dose FOFB group; D: middle-dose FOFB group; E: high-dose FOFB group; F: prednisone group.

**Figure 5:**
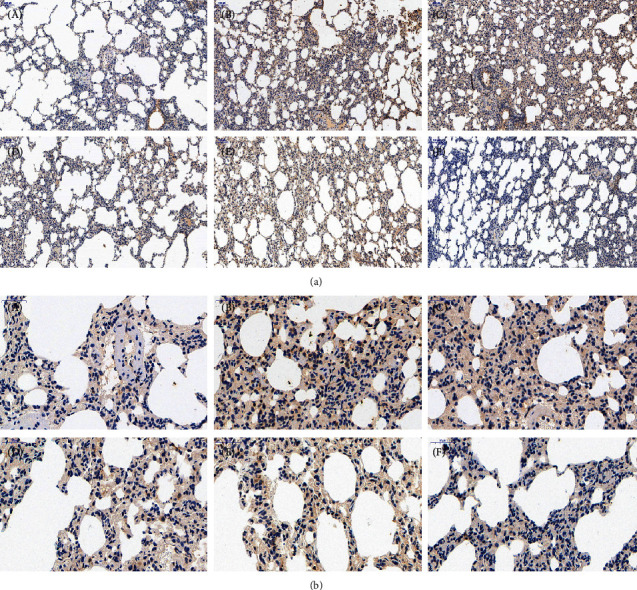
Observation of p-STAT1 by immunohistochemical staining ((a):100x; (b):400x). A: blank (control) group; B: BLM group; C: low-dose FOFB group; D: middle-dose FOFB group; E: high-dose FOFB group; F: prednisone group.

**Figure 6:**
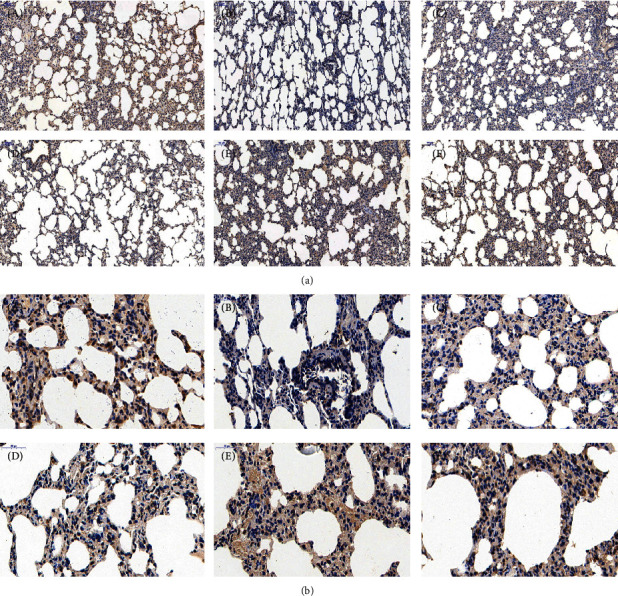
Observation of SOCS3 by immunohistochemical staining ((a):100x; (b):400x). A: blank (control) group; B: BLM group; C: low-dose FOFB group; D: middle-dose FOFB group; E: high-dose FOFB group; F: prednisone group.

**Figure 7:**
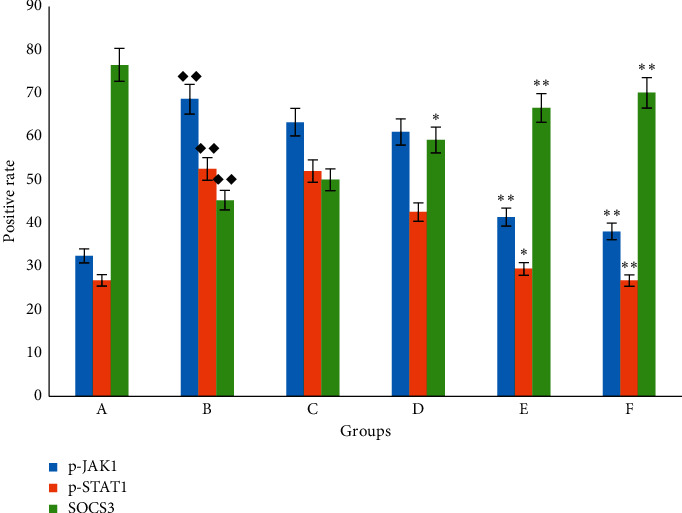
The positive rate of p-JAK1, p-STAT1, and SOCS3 with immunohistochemical staining. A: blank (control) group; B: BLM group; C: low-dose FOFB group; D: middle-dose FOFB group; E: high-dose FOFB group; F: prednisone group. Compared with blank group ◆*p* < 0.05, ◆◆*p* < 0.01; compared with the BLM group ^*∗*^*p* < 0.05, ^*∗∗*^*p* < 0.01; compared with the prednisone acetate group #*p* < 0.05, ##*p* < 0.01.

The activated p-JAK1/p-STAT1 protein is closely related to the occurrence of inflammation and studies have shown that these two proteins are also abnormal expression in IPF. SOCS3 is the most recognized negative regulator of this pathway. As shown in Figures 4–6, in the control group, immunostaining could be observed in tracheal and vascular wall, and interstitial area was exceedingly weakly positive. In contrast, SOCS3 staining in these sites was strongly positive. Compared with the blank group, in the BLM group, immunostaining of p-JAK and p-STAT1 was strongly positive (*p* < 0.01); SOCS3 staining was weakly positive (*p* < 0.01). The positive rate expression of each treatment group was between the above two groups, and there was no significant difference between the FOFB high-dose group and the prednisone acetate group and the normal group (Figure 7).

### 3.4. Effects of FOFB on the Expression of SOCS3 with IPF in Rats

**Figure 8:**
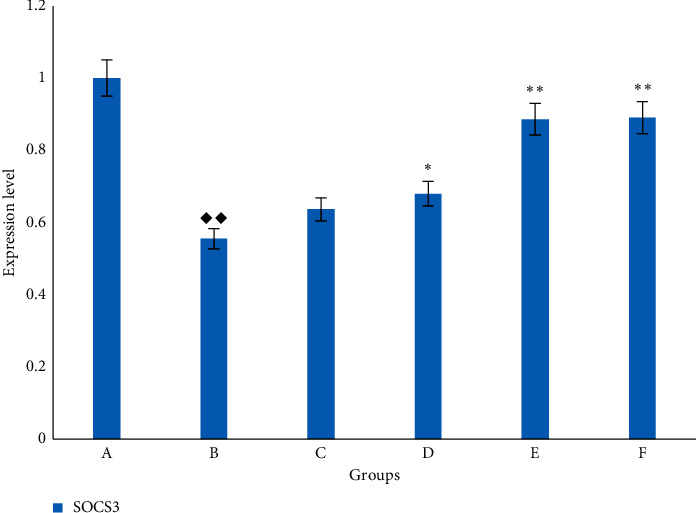
The expression of SOCS3 mRNA in rat lung. A. blank (control) group; B: BLM group; C: low-dose FOFB group; D: middle-dose FOFB group; E: high-dose FOFB group; F: prednisone group. Compared with blank group ◆*p* < 0.05, ◆◆*p* < 0.01; compared with the BLM group ^*∗*^*p* < 0.05, ^*∗∗*^*p* < 0.01; compared with the prednisone acetate group #*p* < 0.05, ##*p* < 0.01.

RT-PCR reflected that the mRNA expression of SOCS3 was significantly lower in the lung tissue of the model group than that of the blank group (*p* < 0.05). The results are shown in Figure 8.

## 4. Discussion

IPF is an interstitial lung disease with high mortality and poor prognosis [19]. Researchers have found that inflammation was closely related to its occurrence and development [8, 20]. JAK1/STAT1 is a key signaling pathway to regulate inflammation [8]. At the same time, studies have shown that JAK1 and STAT1 are abnormally expressed in the lung tissue of IPF mice [8]. SOCS3 is a negative regulator of JAK1/STAT1 and can block the conduction of this signal pathway [13]. In recent years, Traditional Chinese Medicine plays an important role in the treatment of IPF with its unique advantages and efficacy [21]. *Oxytropis falcata* Bunge is one of the three anti-inflammatory drugs of Tibetan medicine, among which flavonoids extract has the strongest anti-inflammatory activity [15]. It has been confirmed that FOFB can reduce the expression of some profibrotic factors and slow down the process of renal interstitial fibrosis [18]. Based on the above theory, we investigated the effect of FOFB on the inflammation-induced lung tissue damage, the degree of fibrosis, and the expression of p-JAK1, p-STAT1, and SOCS3.

Intratracheal injection of BLM to establish the IPF model is the most classic method because it is similar to the pathological process of human pulmonary fibrosis [22]. In our study, observing the pathological morphology of lung tissue of BLM-induced IPF rats by HE and Masson staining, we found FOFB group could improve the degree of alveolitis and delay the progress of IPF. The existing research has confirmed that p-JAK1 and p-STAT1 were involved in the formation of IPF. JAK1 is inactive before exposure to cytokines, but the binding of cytokines to their receptors induces their own activation through transphosphorylation. Upon activation, phosphorylated JAK1, named p-JAK1, serve as docking sites for members of the STAT transcription factor family. Receptor-localized STAT1 is then phosphorylated by p-JAK1, named p-STAT1, causing a biochemical cascade [14]. Through the results of immunohistochemistry and RT-PCR, we found that the FOFB group could increase the expression of SOCS3 protein and further reduce the expression of p-JAK1 and p-STAT1 to play a therapeutic role.

## 5. Conclusion

FOFB has a therapeutic effect on inflammation-mediated idiopathic pulmonary fibrosis, and its mechanism may be to inhibit the expression of p-JAK1 and p-STAT1 inflammatory proteins by upregulating the expression of SOCS3.

## Figures and Tables

**Table 1:** Lung HE staining score of rats in each group.

Groups	Staining score	Comparison with blank group	Comparison with BLM group	Comparison with prednisone acetate group	Comparison among groups
	*M* (*Q*_1_, *Q*_3_)	*p*	*p*	*p*	*p*
Blank group	1.00 (1.00, 1.00)	—	<0.0001	0.98	

BLM group	3.00 (3.00, 3.00)	<0.0001	—	0.001	

Low-dose group of FOF	3.00 (2.00, 3.00)	0.005	0.788	0.004	

Middle-dose group of FOF	2.00 (2.00, 2.25)	0.008	0.036	0.021	

High-dose group of FOF	1.00 (1.00, 2.00)	0.788	0.005	1.000	

Prednisone acetate group	1.00 (1.00, 1.25)	0.980	0.001	—	

**Table 2:** Lung Masson staining score of rats in each group.

Groups	Staining score	Comparison with blank group	Comparison with BLM group	Comparison with prednisone acetate group	Comparison among groups
	$\bar{x}$ ± *S*	*p*	*p*	*p*	*p*
Blank group	0.6667 ± 0.8165	—	<0.001	0.204	

BLM group	3.5000 ± 0.5477	<0.001	—	<0.001	

Low-dose group of FOF	3.1667 ± 0.4082	<0.001	0.393	<0.001	

Middle-dose group of FOF	2.1667 ± 0.4082	0.001	0.002	0.014	

High-dose group of FOF	1.0000 ± 0.8944	0.393	<0.001	0.668	

Prednisone acetate group	1.1667 ± 0.7527	0.204	<0.001	—	

## Data Availability

All the data related to this article are described as histopathological pictures and statistical analysis in the manuscript.

## Supplementary Materials

Supplementary Materials Szapiel's semiquantitative grading system.
