# Supplementary material for: Effect of Total Flavonoids of Oxytropis falcata Bunge on the Expression of p-JAK1-and p-STAT1-Related Proteins in Idiopathic Pulmonary Fibrosis
Source: Evid Based Complement Alternat Med. 2020 Aug 28;2020:2407239. doi: 10.1155/2020/2407239 (PMC7474768; doi:10.1155/2020/2407239)
Supplement: Supplementary Materials — Szapiel's semiquantitative grading system. [file 2407239.f1.doc]

Szapiel’s semiquantitative grading system

| Level | Alveolitis | Integral value | Level | Pulmonary fibrosis | Integral value |
| --- | --- | --- | --- | --- | --- |
| - | noalveolitis or a small amount of inflammatory cells | 0 | - | No pulmonary fibrosis | 0 |
| + | mild alveolitis，mononuclear cells are clear，the alveolar interval is widened， and the lesion range is limited to less than 20% of the whole lung | 1 | + | mild pulmonary fibrosis，the lesion range is less than 20% of the whole lung | 1 |
| ++ | moderate alveolitis， disease range of the whole lung 21% ～ 50% | 2 | ++ | moderate pulmonary fibrosis，the lesion ranges from 21% to 50% of the whole lung，and alveolar dysfunction | 2 |
| +++ | severe alveolitis，diffuse alveolar disease with a lesion range of more than 51% | 3 | +++ | severe pulmonary fibrosis，the lesion range was greater than 51% with alveo- lar fusion and obvious disorder of pulmonary parenchyma | 3 |
